# Emergence of carbapenem-resistant *Salmonella* Mbandaka through IS*26*-driven *bla*_NDM-1_ mobilization and chromosomal structural variation

**DOI:** 10.1128/spectrum.00967-25

**Published:** 2025-08-15

**Authors:** Huijuan Song, Siyu Zou, Yue Wang, Zhongju Chen, Ziyong Sun, Cui Jian

**Affiliations:** 1 Department of Laboratory Medicine, Tongji Hospital, Tongji Medical College, Huazhong University of Science and Technology 12443, Wuhan, China; University of Kentucky, Lexington, Kentucky, USA

**Keywords:** *bla*_NDM-1_, *Salmonella*, IS*26*, whole-genome sequencing, amplification, carbapenem resistance

## Abstract

*Salmonella enterica* subsp. *enterica* serovar Mbandaka (*S*. Mbandaka) represents an emerging public health threat due to its remarkable genomic adaptability, involvement in multinational foodborne outbreaks, and the ability to establish persistent infections. Here, we report the first documented isolation of a carbapenem-resistant *S*. Mbandaka strain, a significant public health concern given the critical role of carbapenems in treating multidrug-resistant infections. Five clonal *S*. Mbandaka isolates were obtained from a single patient, with three isolates (SM_F22R, SM_F28R, and SM_B30R) exhibiting carbapenem resistance mediated by the *bla*_NDM-1_. The comprehensive genomic analysis uncovered a 1,220 kb chromosomal inversion mediated by IS*26* through intramolecular replicative transposition (in *trans*), generating the SM_F28R and SM_B30R variants. Using S1 pulsed-field gel electrophoresis, Southern blotting, and conjugation experiments, we demonstrated the transfer of *bla*_NDM-1_: chromosomal localization in SM_F22R and plasmid-borne (IncC-type) carriage in SM_F28R and SM_B30R. Notably, the region carrying *bla*_NDM-1_ in both locations was flanked by IS*26* and completely identical, indicating that IS*26* may facilitate the transfer of *bla*_NDM-1_ between the chromosome and plasmid. Additionally, we detected the amplification of *mph(A*) on the chromosome and *bla*_NDM-1_ on the IncC plasmid. The expression level of *bla*_NDM-1_ in SM_F22R was lower than that in SM_F28R and SM_B30R, consistent with SM_F22R exhibiting lower carbapenem resistance compared to SM_F28R and SM_B30R. This study reported the IS*26*-mediated transfer of *bla*_NDM-1_ between the chromosome and a plasmid in *S*. Mbandaka, highlighting the critical role of transposable elements in disseminating carbapenem resistance.

**IMPORTANCE:**

As a clinically significant foodborne pathogen, carbapenem-resistant *Salmonella* presents a significant therapeutic challenge due to its extensive antibiotic resistance. While *bla*_NDM-1_ is conventionally plasmid-borne, our study elucidates its transfer between chromosomes and a conjugative IncC plasmid in *Salmonella*. Our findings demonstrate the pivotal involvement of IS*26* and IS*CR1* mobile genetic elements in orchestrating genomic rearrangements while concurrently mediating the horizontal transfer and subsequent amplification of antimicrobial resistance determinants. This study provides an important theoretical basis for an in-depth analysis of the horizontal transfer mechanism of bacterial resistance genes.

## INTRODUCTION

*Salmonella* is a major zoonotic foodborne pathogen that imposes substantial disease burdens on both human and animal populations (1). According to data from the Institute for Health Metrics and Evaluation, invasive non-typhoidal *Salmonella* infections were responsible for an estimated 594,000 cases globally in 2019, resulting in 79,000 deaths and 6.11 million disability-adjusted life years (2). They disproportionately affect pediatric populations, with infants demonstrating particular susceptibility (3–7).

Among various serovars, *Salmonella enterica* subsp. *enterica* serovar Mbandaka (*S*. Mbandaka) has emerged as an increasingly significant pathogen despite its relatively lower prevalence compared to *Salmonella* Enteritidis or *Salmonella* Typhimurium (8). Its strong association with poultry and livestock reservoirs (9, 10), combined with its ranking among Europe’s top ten human salmonellosis-causing serovars (9), underscores its public health importance. Notably, *S*. Mbandaka has been implicated in multistate outbreaks in the United States (11) and exhibited concerning dissemination patterns in Chinese poultry farms and healthcare settings (12, 13). Clinical evidence further identified *S*. Mbandaka as significantly associated with persistent infections (14).

Current treatment guidelines recommend third-generation cephalosporins or azithromycin as empirical therapy for pediatric salmonellosis (15). However, the overuse of antibiotics has led to increases in multidrug-resistant (MDR) *Salmonella*, posing a grave threat to public health (16–18). As the last-line agents for MDR Gram-negative bacterial infections (19), the emergence of carbapenem resistance in *Salmonella* demands special attention, despite its relatively low prevalence (20–22). The identified carbapenem resistance mechanisms in *Salmonella* include (i) carbapenemase production, such as New Delhi metallo-beta-lactamase (NDM) and (ii) overexpression of extended-spectrum β-lactamase or AmpC β-lactamase combined with the loss of outer membrane porins (20–22). These findings underscore the urgent need for resistance mechanism studies to develop effective containment strategies.

Pan-genome analyses have demonstrated that *S*. Mbandaka exhibits remarkable genomic adaptability through recurrent gene acquisition and loss events, particularly involving virulence factors and antimicrobial resistance determinants, which may pose emerging public health threats (23). Plasmid-mediated horizontal gene transfer, predominantly via incompatibility group plasmids (IncHI2A and IncHI2), drives the rapid spread of resistance genes among *S*. Mbandaka populations (23). Additionally, the insertion sequence 26 (IS*26*) mainly plays an important role in the spread of antimicrobial resistance via two distinct transposition mechanisms (24, 25): (i) replicative transposition, which generates DNA inversions (in *trans*) or deletions (in *cis*) while producing characteristic 8 bp target site duplications (TSDs) and additional IS*26* copies, and (ii) conservative transposition, which targets existing IS*26* elements without TSDs or IS*26* replication. Through these processes, IS*26* facilitated resistance gene amplification through tandem duplication and transfer of resistance gene cassettes (26).

Here, we reported the first case of carbapenem-resistant *S*. Mbandaka. By comparing antimicrobial susceptibility profiles and genetic variations, including gene rearrangement, horizontal gene transfer, and gene amplification, among five *S*. Mbandaka isolates from a single patient, this study aimed to explore mechanisms of carbapenem resistance development and dissemination *in vivo*, revealing bacterial adaptation to host environmental pressures during infection and providing valuable insights for resistance containment.

## MATERIALS AND METHODS

### Bacterial identification and subpopulation isolation

A total of five isolates were used in this study: SM_F22S/SM_F22R from a fecal sample collected on December 22, SM_F28S/SM_F28R from a fecal sample collected on December 28, and SM_B30R from a blood culture obtained on December 30. All isolates were from a 13-month-old patient who presented with fever and diarrhea at Tongji Hospital on December 21, 2021. The fecal specimen collected on December 22, 2021, yielded characteristic colonies after 24-hour incubation at 37°C: colorless colonies on MacConkey agar and black-centered colonies with transparent margins on xylose lysine deoxycholate (XLD) agar without any antibiotics. Initial identification was confirmed using the Autof ms1000 matrix-assisted laser desorption/ionization time-of-flight mass spectrometry system (Autobio Diagnostics, Zhengzhou, China), which reliably identified all isolates as *Salmonella*. Serotyping using commercial antisera (Statens Serum Institut Diagnostica, Denmark), according to the White-Kauffmann-Le Minor scheme (27), revealed the antigenic formula O:7, H:z10 (phase 1). The initially absent phase 2 flagellar antigen (z15) was subsequently induced using SG1 serum, and the serotype was determined to be *S*. Mbandaka (O7:z10:z15).

For each specimen, two to three typical *Salmonella* colonies on XLD agar were subcultured onto blood agar plates without any antibiotics using a three-zone streaking method and incubated at 37°C for 18–24 hours (28). After subculturing, 0.5 McFarland bacterial suspensions were prepared using two to three morphologically uniform pure colonies isolated from blood agar plates and further subjected to Kirby-Bauer disk diffusion using imipenem (10 µg) and meropenem (10 µg) disks (28). Intriguingly, the *S*. Mbandaka isolate obtained on December 22 exhibited a distinctive double inhibition zone pattern, characterized by an outer susceptible zone (>23 mm diameter) and an inner resistant zone (<19 mm diameter) (Fig. S1). We isolated pure cultures from both the inner and outer peripheries of double inhibition zones through successive passages on blood agar, establishing stable phenotypic variants designated as carbapenem-susceptible (SM_F22S) and carbapenem-resistant (SM_F22R) populations. This isolation strategy was replicated for the December 28 fecal specimen, yielding analogous carbapenem-susceptible (SM_F28S) and carbapenem-resistant (SM_F28R) subpopulations (Fig. S1). In contrast, the blood culture isolates (SM_B30R) on December 30 demonstrated monomorphic carbapenem resistance (6 mm inhibition zone) without evidence of susceptible subpopulations.

### Antimicrobial susceptibility testing (AST)

The minimum inhibitory concentrations (MICs) of five clinical isolates (SM_F22S, SM_F22R, SM_F28S, SM_F28R, and SM_B30R) were determined by the broth microdilution method (28). The following antimicrobial agents and concentration ranges were evaluated: colistin (0.06–128 mg/L) and polymyxin B (0.06–32 mg/L), with susceptibility interpretations based on the European Committee on Antimicrobial Susceptibility Testing (https://www.eucast.org/) guidelines. Eravacycline (0.06–128 mg/L) and tigecycline (0.06–32 mg/L) were interpreted using the Food and Drug Administration breakpoints (https://www.fda.gov/). We further evaluated imipenem (IPM, 0.06–128 mg/L), meropenem (MEM, 0.06–128 mg/L), imipenem-relebactam (IMR, 0.06/4–128/4 mg/L), meropenem-vaborbactam (MEV, 0.06/8–128/8 mg/L), ceftazidime (0.06–128 mg/L), ceftazidime-avibactam (0.06/4–128/4 mg/L), amikacin (0.06–128 mg/L), cefepime (0.06–128 mg/L), aztreonam (0.06–128 mg/L), ciprofloxacin (0.06–128 mg/L), trimethoprim-sulfamethoxazole (0.06/1.14–128/2432 mg/L), and azithromycin (0.06–128 mg/L), applying the Clinical and Laboratory Standards Institute M100 Ed34 guideline for interpretation (28). Notably, the breakpoint for aztreonam-avibactam (0.06/4–128/4 mg/L) was referenced to aztreonam (29). The *S*. Mbandaka susceptibility test results for azithromycin were interpreted based on the breakpoints for *Salmonella* Typhi. *Escherichia coli* (*E. coli*) ATCC 25922 and *Pseudomonas aeruginosa* ATCC 27853 were employed for quality control assessment.

### Plasmid conjugation assay

Rifampin-resistant *E. coli* C600 served as the recipient, while carbapenem-resistant *Salmonella* isolates acted as donors in broth mating experiments to investigate the transferability of carbapenem resistance (30). The donor and recipient strains were incubated at 37°C in Luria-Bertani (LB) broth with shaking at 200 rpm until the logarithmic phase of growth. The donor and recipient cells were mixed at a 1:3 volumetric ratio (20 µL donor: 60 µL recipient) in fresh LB medium and incubated for 4 hours at 37°C under static conditions to facilitate plasmid transfer. Post-mating, 50 µL of the conjugation mixture was diluted in 100 µL LB broth and plated on selective media containing meropenem (2 µg/mL) and rifampin (500 µg/mL) to isolate transconjugants. Successful conjugation events were confirmed by PCR amplification of carbapenemase genes and AST of the transconjugants.

### Molecular typing and resistance gene localization

Pulsed-field gel electrophoresis (PFGE) was performed to assess the clonal relatedness among isolates (31). Genomic DNA embedded in 1% agarose plugs was digested with XbaI restriction enzyme (37°C for 4 hours) and separated using the CHEF Mapper XA system (Bio-Rad Laboratories, Hercules, CA, USA) under optimized electrophoretic conditions (6 V/cm, 14°C, with pulse times ramping from 2.16 to 63.8 seconds over 19 hours). PFGE banding patterns were analyzed with BioNumerics v8.0 (Applied Maths NV, Sint-Martens-Latem, Belgium), with isolate similarity calculated using Dice coefficient (1.0% optimization and 1.5% tolerance) and unweighted pair group method with arithmetic mean cluster analysis. Isolates were categorized according to Tenover criteria as follows: indistinguishable (identical band patterns), closely related (1–3 band differences indicating clonal microevolution), or distinct (≥4 band differences representing unrelated clones) (32).

For localization of *bla*_NDM-1_, PCR-confirmed *bla*_NDM-1_-positive isolates were subjected to S1-PFGE coupled with Southern hybridization analysis (33). The *bla*_NDM-1_-positive PCR amplicon was labeled using the digoxigenin (DIG) High Prime DNA Labeling Kit I (Roche Diagnostics, Mannheim, Germany) to generate a highly specific DIG-labeled *bla*_NDM-1_ probe. Genomic DNA prepared in agarose blocks was digested with S1 nuclease (Takara Bio Inc., Shiga, Japan) at 23°C for 40 minutes to achieve plasmid linearization. The PFGE-separated DNA fragments were transferred to nylon membranes and hybridized with DIG-labeled *bla*_NDM-1_ probes, allowing simultaneous plasmid sizing via reference markers and precise *bla*_NDM-1_ localization through chemiluminescent detection.

### Whole-genome sequencing and bioinformatics analysis

Genomic DNA was extracted from single-colony overnight cultures using the SDS method (34), with quality assessment performed via: (1) 1% agarose gel electrophoresis confirming intact high-molecular-weight DNA (2), quantification using Qubit 3.0 Fluorometer (Thermo Fisher Scientific, Waltham, MA, USA; ≥70 ng/µL), and (3) purity evaluation by NanoDrop spectrophotometry (Thermo Fisher Scientific, Waltham, MA, USA; A260/A280 ratio 1.8–2.0).

For Illumina sequencing, genomic DNA was fragmented to 350 bp using a Covaris ultrasonicator (Covaris, Woburn, MA, USA), followed by library preparation with the NEBNext Ultra DNA Library Prep Kit (New England Biolabs, Ipswich, MA, USA). Libraries were purified using AMPure XP beads (Beckman Coulter Life Sciences, Indianapolis, IN, USA), analyzed for size distribution with Agilent 2100 Bioanalyzer (Agilent Technologies, Santa Clara, CA, USA), quantified via quantitative real-time PCR (qPCR), and sequenced on NovaSeq 6000 platform (Illumina, Inc., San Diego, CA, USA) with 2 × 150 bp paired-end reads. In parallel, approximately 10 kb DNA fragments were selected using the BluePippin system (Sage Science, Beverly, MA, USA), followed by end repair and adapter ligation with SQK-LSK109 kit (Oxford Nanopore Technologies plc, Oxford, UK) for sequencing on PromethION platform (Oxford Nanopore Technologies, Oxford, UK).

The bioinformatics analysis included quality filtering of raw reads (Fastp, https://github.com/OpenGene/fastp, v0.23.2), assembly, and polishing (Unicycler, https://github.com/rrwick/Unicycler, v0.4.5) to obtain the complete genome sequences. The multilocus sequence typing (MLST) analysis was performed using the 7-gene scheme (*aroC, dnaN, hemD, hisD, purE, sucA,* and *thrA*) for *Salmonella enterica,* with sequence type (ST) determination conducted through the EnteroBase platform (https://enterobase.warwick.ac.uk/). Genome annotation was conducted using Prokka (https://github.com/tseemann/prokka, v1.14.6), while ISs were annotated via ISfinder (https://www-is.biotoul.fr/blast.php). The plasmid incompatibility group types and antibiotic resistance genes (ARGs) were identified using ABRicate (https://github.com/tseemann/abricate, v2.0.0) with the PlasmidFinder (v2.1.0), ResFinder (v4.4.3), and CARD (v4.0.0) databases, applying consistent thresholds of ≥90% nucleotide identity and ≥60% coverage. Comparative genomic analysis and visualization were conducted using Easyfig (http://mjsull.github.io/Easyfig/, v2.2.5), with read mapping depth analysis performed as described previously (33).

### Quantitative real-time PCR (qPCR)

The copy number and expression level of *bla*_NDM-1_ were quantified using qPCR with three biological replicates and three technical replicates for each sample, following the procedures and primers as described previously (33). Statistical analysis was conducted using the independent sample *t*-test, with a statistical significance threshold set at *P* < 0.05.

## RESULTS

### Clinical course and microbiological findings

A 13-month-old patient initially presented at Zhijiang City People’s Hospital on November 11, 2021, with right cervical lymphadenopathy and persistent fever. The antimicrobial regimens, including ceftezole (2 days), cefoperazone/sulbactam (1 day), azithromycin (1 day), and methylprednisolone (1 day), failed to control disease progression, prompting transfer to Yichang Central Hospital on November 15. The patient subsequently developed a maculopapular rash on bilateral lower extremities and generalized lymphadenopathy. Despite switching to therapy with cefotaxime, azithromycin, and intravenous immunoglobulin, febrile episodes persisted, necessitating transfer to Wuhan Children’s Hospital on November 20. There, antimicrobial therapy was adjusted to ceftezole (November 20), meropenem (November 21–27) with teicoplanin (November 21–22), and vancomycin (November 23–28), achieving transient clinical improvement until symptom recurrence upon discharge, characterized by vomiting, diarrhea, and fever.

Following presentation with a generalized rash and *Salmonella*-positive stool cultures at Yichang Third People’s Hospital on December 17, the patient was transferred to Tongji Hospital, Huazhong University of Science and Technology, on December 21 due to disease complexity (Fig. S1). Upon admission, the patient exhibited persistent diarrhea (two to three episodes/day of yellow, loose stools). Microbiological analysis of stool samples collected on December 22 and 28 revealed co-existing carbapenem-susceptible and carbapenem-resistant *Salmonella* subpopulations. While initial clinical improvement was observed following antimicrobial therapy (Fig. S1), subsequent deterioration manifested on December 30 with recrudescent fever (39.6  °C) and isolation of carbapenem-resistant *Salmonella* from bloodstream cultures. Systemic inflammatory markers were elevated, including white blood cells (15.68 × 10⁹/L), neutrophils (10.64 × 10⁹/L), IL-6 (13.51 pg/mL), ferritin (1089.8 µg/L), procalcitonin (2.6 ng/mL), and C-reactive protein (90.3 mg/L), collectively confirming a sepsis diagnosis. The clinical course was further complicated by secondary infections, including pulmonary aspergillosis (*Aspergillus fumigatus* isolated from bronchoalveolar lavage fluid on January 12 and 14) and urinary tract infection (*Enterococcus faecium* from urine cultures on January 17). During hospitalization, targeted antimicrobial therapy was implemented, including biapenem, aztreonam, ceftazidime/avibactam, cefoperazone/sulbactam, trimethoprim/sulfamethoxazole, levofloxacin, teicoplanin, micafungin, and voriconazole, with detailed treatment timelines illustrated in Fig. S1. After symptomatic supportive treatment, such as anti-infection, partial microbiological clearance was achieved, followed by negative blood and fecal cultures. However, multiorgan dysfunction remained evident at the time of the family-requested discharge on February 7, 2022.

### Chromosomal sequence variations among isolates

#### Five *S*. Mbandaka isolates belonged to a clonal lineage

Five *S*. Mbandaka isolates (SM_F22Rchr, CP117850.2; SM_F22Schr, CP117299.2; SM_F28Schr, CP117181.2; SM_F28Rchr, CP138307.1; SM_B30Rchr, CP138305.1) were identified as ST413 through alignment with the EnteroBase database. PFGE with XbaI digestion demonstrated that isolates SM_F22S, SM_F28S, SM_F28R, and SM_B30R shared indistinguishable banding patterns (100% similarity), while SM_F22R showed 94.1% similarity, differing by an additional 210 kb band and the absence of a 33 kb fragment (Fig. S2A and B).

#### IS*26*-mediated chromosomal inversion

**Fig 1:**
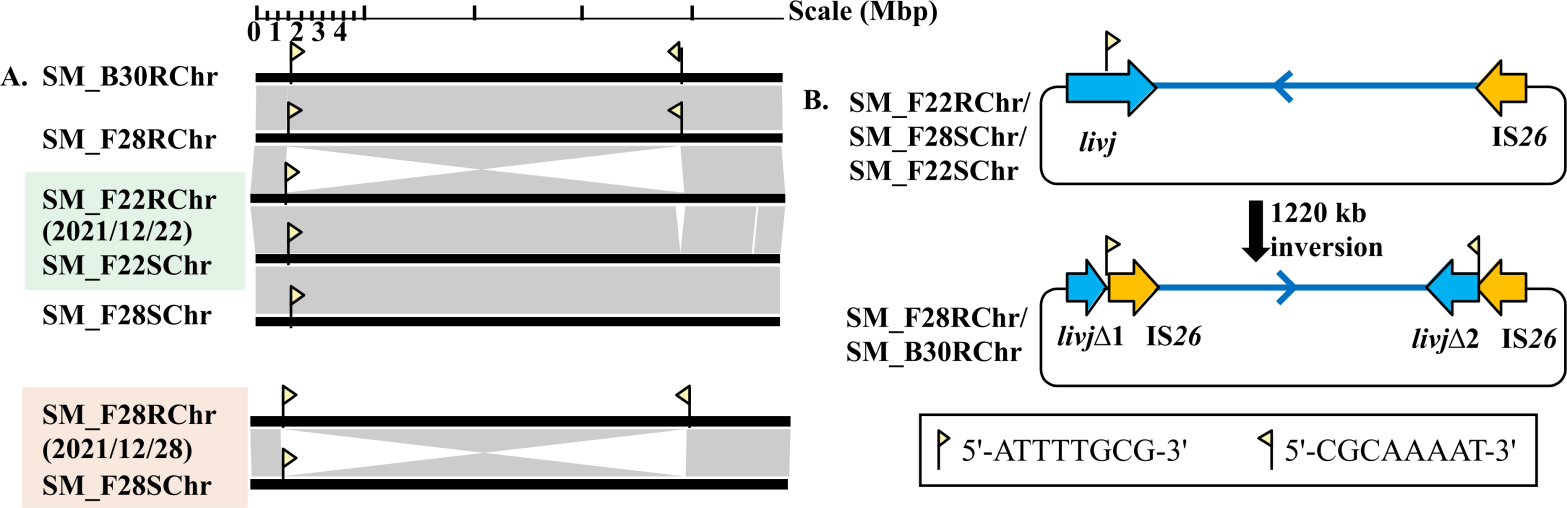
Whole-chromosome alignment of five *Salmonella* Mbandaka isolates. (**A**) The sequence alignment results of five chromosomal sequences (drawn to scale). Chromosome lengths vary as follows: SM_F22S: 4,839,259 bp; SM_F22R: 4,931,346 bp; SM_F28S: 4,839,261 bp; SM_F28R: 4,861,942 bp; and SM_B30R: 4,848,834 bp. SM_F28R and SM_B30R displayed an inversion of a ~ 1,220 kb chromosomal segment relative to its susceptible counterpart SM_F28S (coordinates 284,920–3,909,306 bp). Gray shading marks 100% nucleotide identity between sequences. (**B**) Schematic representation of IS*26*-mediated DNA inversion through intramolecular transposition (in *trans*). The blue arrow indicates the 1,220 kb inverted region. The diagram is not to scale. The yellow flag facing right represents the 8 bp sequence 5′-ATTTTGCG-3′, while the yellow flag facing left represents the 8 bp sequence 5′-CGCAAAAT-3′.

Whole-genome alignment of the five isolates demonstrated close phylogenetic relationships between the chromosomes of SM_F22S and SM_F28S, as well as between those of SM_F28R and SM_B30R (Fig. 1A), while SM_F22S/SM_F22R from the same fecal specimen showed significant chromosomal similarity. Notably, SM_F28R and SM_B30R displayed an inversion of a ~ 1,220 kb chromosomal segment relative to its susceptible counterpart SM_F28S (coordinates 284,920–3,909,306 bp) (Fig. 1A; Fig. S3). Chromosomal comparisons identified a 1,220 kb genomic segment flanked by an IS*26* copy and the intact *livJ* gene containing the 8 bp motif 5′-ATTTTGCG-3′ in SM_F22S, SM_F28S, and SM_F22R (Fig. 1B; Fig. S3). On the chromosomes of SM_F28R and SM_B30R, the IS*26* transposase precisely recognizes an 8 bp motif (5′-ATTTTGCG-3′) within the *livJ* gene, resulting in truncation of *livJ* at this site and inversion between the *livJ*-IS*26* sequences through intramolecular replicative transposition. Furthermore, additional IS*26* copies were generated, with two IS*26* copies in reverse orientation. After intramolecular transposition in *trans*, the 8 bp motif (5′-ATTTTGCG-3′) is presented as reverse complements (5′-CGCAAAAT-3′) of each other adjacent to one end of an IS*26* copy (Fig. 1B; Fig. S3).

#### Identification of *bla*_NDM-1_ and amplification of *mph*(A) on SM_F22R chromosome

Comparative genomic analysis revealed that the SM_F22R chromosome harbored a ~ 26 kb MDR region integrated between 3.9 and 4.0 Mbp, which was absent in the other four isolates. This region carried *bla*_NDM-1_, *dfrA12*, *sul1*, *ble*, *msr*(E), and *mph*(E) genes, bracketed by a 5,193 bp sequence (IS*26-mph*(A)-*mrx*(A)-*mphR*(A)-IS*26*) oriented in the same direction. This structure, subsequently registered as pTn*7682* by the Transposon Registry, was present as a single copy in the chromosomes of SM_F22S and SM_F28S. In contrast, tandem copies of pTn*7682* were detected in SM_F22R, SM_F28R, and SM_B30R chromosomes, with SM_F22R exhibiting an exceptionally high copy number (~20 copies). In summary, three chromosomal variations were observed in SM_F22R compared to SM_F28R and SM_B30R: a 26 kb MDR region carrying *bla*_NDM-1_, a 1,220 bp chromosomal inversion, and an exceptionally high copy number of pTn*7682*. Chromosomal length variation among isolates primarily resulted from the integration of the MDR region and differential pTn*7682* copy number.

XbaI restriction site analysis and genome digestion using SnapGene software demonstrated that the SM_F22R chromosome yielded a unique ~130 kb fragment after XbaI digestion, accompanied by deletions of ~30 kb and ~180 kb bands, consistent with the XbaI-PFGE banding pattern (Fig. S3).

### Antimicrobial susceptibility and resistance gene profiles

**Fig 2:**
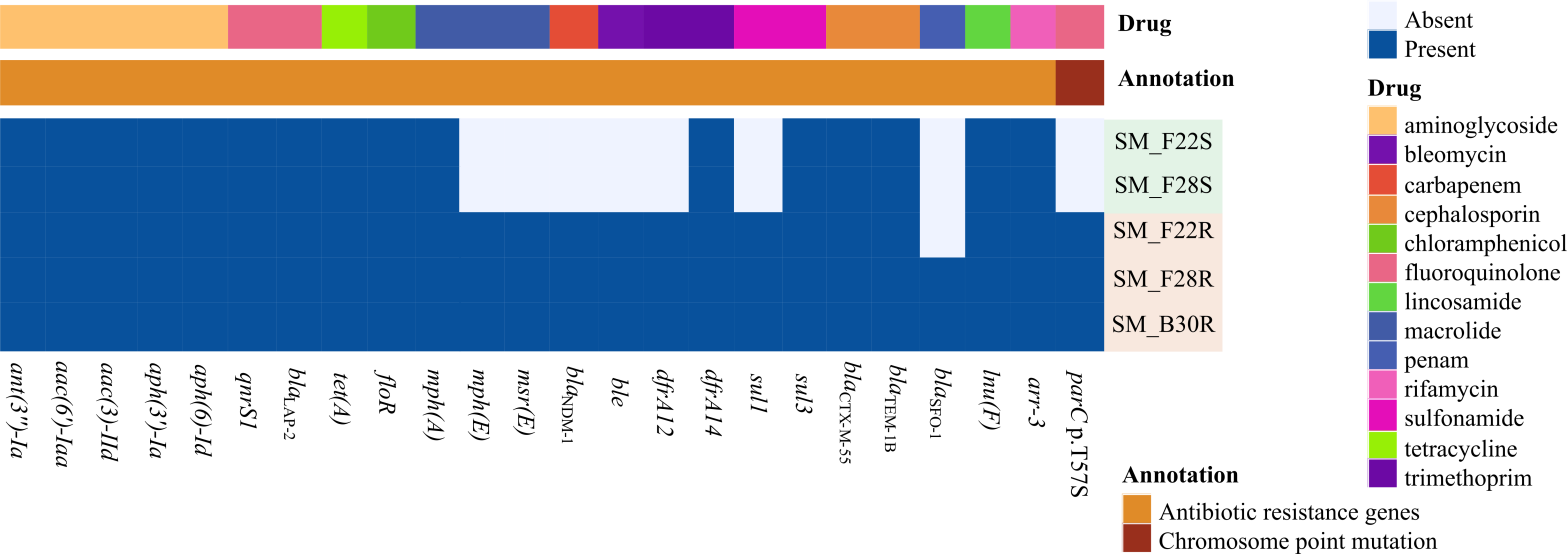
Distribution of antimicrobial resistance genes of five *Salmonella* Mbandaka isolates. The *aac(6')-Iaa* gene is an intrinsic gene in *Salmonella*.

The MICs for antibiotics are presented in Table 1. All five isolates exhibited high-level azithromycin resistance (MIC >128 mg/L), regardless of *mph*(A) copy numbers. SM_F22S and SM_F28S remained susceptible to carbapenems (IPM and MEM) as well as novel β-lactamase inhibitor combinations (IMR, MEV, and ceftazidime-avibactam) and trimethoprim-sulfamethoxazole. In stark contrast, their resistant counterparts (SM_F22R and SM_F28R), along with SM_B30R, displayed resistance to these agents (Table 1), correlating with the acquisition of ARGs (*bla*_NDM-1_, *dfrA12*, *sul1*, *ble*, *msr*(E), and *mph*(E); Fig. 2). Notably, SM_F28R and SM_B30R exhibited higher MICs (IPM: 64 mg/L; IMR/MEM/MEV: 128 mg/L) than SM_F22R (IPM: 16 mg/L; IMR: 32 mg/L; MEM/MEV: 64 mg/L).

**TABLE 1:** The minimum inhibitory concentrations (MICs) of antibiotics against the clinical isolates and transconjugants^*a*^

Drug	MIC (mg/L)						
	SM_F22S	SM_F22R	SM_F28S	SM_F28R	SM_B30R	F28R_to_EC600	B30R_to_EC600
IPM	0.25(S)	16(R)	0.25(S)	64(R)	64(R)	64(R)	64(R)
IMR	0.5/4(S)	32/4(R)	0.25/4(S)	128/4(R)	128/4(R)	128/4(R)	128/4(R)
MEM	≤0.06(S)	64(R)	≤0.06(S)	128(R)	128(R)	128(R)	128(R)
MEV	≤0.06/8(S)	64/8(R)	≤0.06/8(S)	128/8(R)	128/8(R)	128/8(R)	128/8(R)
CAZ	64(R)	>128(R)	64(R)	>128(R)	>128(R)	>128(R)	>128(R)
CZA	1/4(S)	>128/4(R)	2/4(S)	>128/4(R)	>128/4(R)	>128/4(R)	>128/4(R)
COL	1(S)	1(S)	1(S)	1(S)	1(S)	1(S)	1(S)
PMB	0.5(S)	0.5(S)	0.5(S)	0.5(S)	0.5(S)	0.5(S)	0.5(S)
AMK	2(S)	1(S)	1(S)	2(S)	1(S)	4(S)	4(S)
FEP	128(R)	>128(R)	128(R)	>128(R)	>128(R)	>128(R)	>128(R)
ATM	>128(R)	>128(R)	>128(R)	>128(R)	>128(R)	32(R)	32(R)
CIP	2(R)	2(R)	2(R)	4(R)	4(R)	0.5(S)	0.5(S)
AZA	0.5/4(S)	0.5/4(S)	0.25/4(S)	1/4(S)	1/4(S)	0.5/4(S)	0.5/4(S)
ERA	8(-)	4(–)	8(–)	8(–)	8(–)	0.5(S)	0.5(S)
SXT	1/19(S)	>128/2432(R)	1/19(S)	>128/2432(R)	>128/2432(R)	>128/2432(R)	>128/2432(R)
TGC	1(S)	2(S)	2(S)	2(S)	2(S)	0.25(S)	0.25(S)
AZM	>128(R)	>128(R)	>128(R)	>128(R)	>128(R)	>128(R)	>128(R)

^*a*^ “–” indicates that the bacterium has no drug sensitivity breakpoint to the drug. F28R_to_EC600 and B30R_to_EC600 refer to *E. coli* EC600 transconjugants harboring plasmids from isolates SM_F28R and SM_B30R, respectively. S, susceptible; R, resistant; IPM, imipenem; IMR, imipenem-relebactam; MEM, meropenem; MEV, meropenem-vaborbactam; CAZ, ceftazidime; CZA, ceftazidime-avibactam; COL, colistin; PMB, polymyxin B; AMK, amikacin; FEP, cefepime; ATM, aztreonam; CIP, ciprofloxacin; AZA, aztreonam-avibactam; ERA, eravacycline; SXT, trimethoprim-sulfamethoxazole; TGC, tigecycline.

### Chromosomal and plasmid localization of *bla*_NDM-1_

Interestingly, while *bla*_NDM-1_ was detected in all carbapenem-resistant isolates, conjugative transfer of resistance was only observed for SM_F28R and SM_B30R (Table 1). Further analysis revealed that *bla*_NDM-1_ in these isolates resided on a transferable ~200 kb IncC plasmid (Fig. S2A and C). However, S1-PFGE and XbaI-PFGE hybridization confirmed the chromosomal integration of *bla*_NDM-1_ in SM_F22R, evidenced by a positive signal corresponding to an extra band of 210 kb (Fig. S2A and C).

### Genetic context and amplification of *bla*_NDM-1_

**Fig 3:**
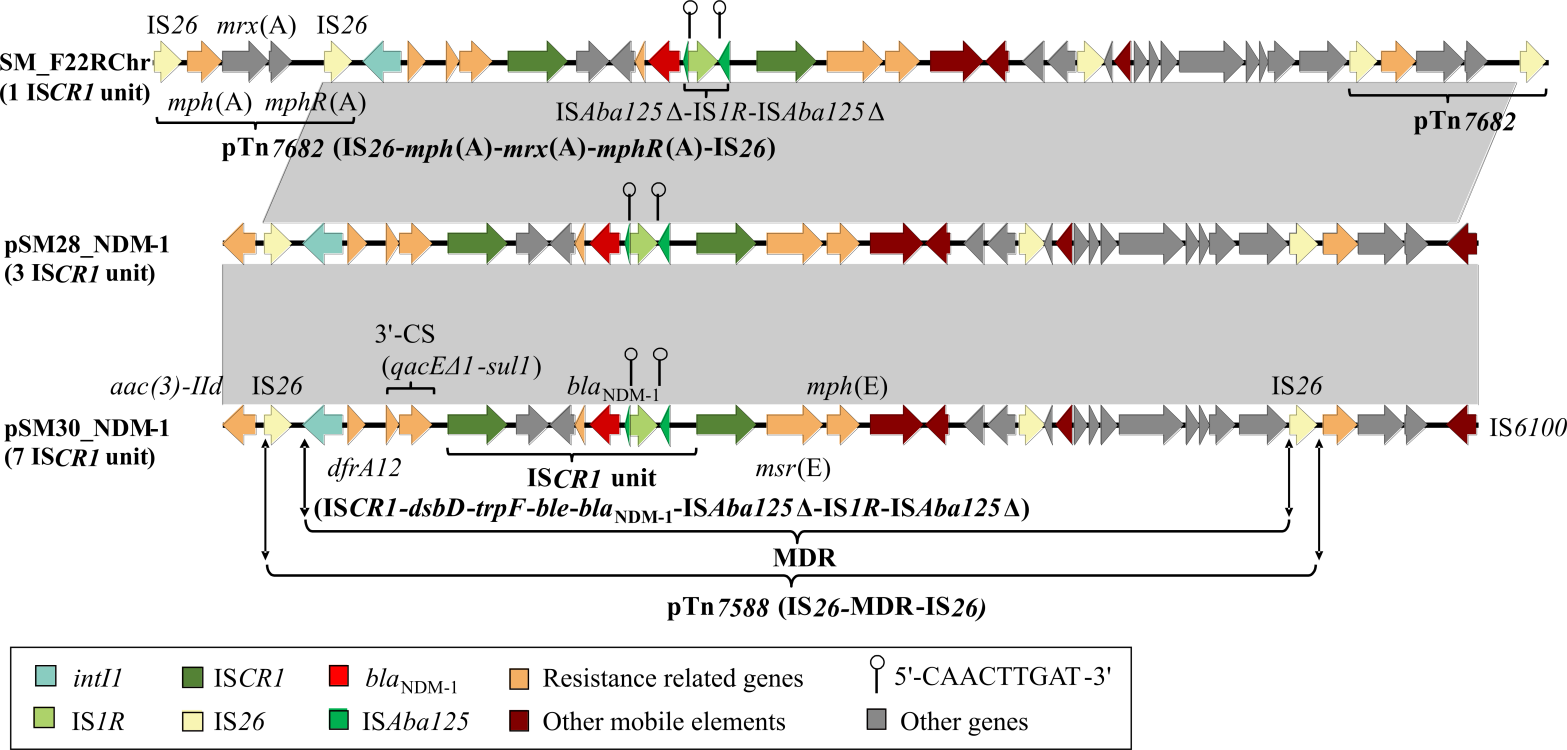
Genetic context of the *bla*_NDM-1_ gene and sequence alignment. IS*1R* was inserted into the truncated IS*Aba125*, forming a 9 bp direct repeat sequence (5′-CAACTTGAT-3′) on the flanks. Genes are represented by colored arrows, with gene names labeled on the graph. The diagram is to scale, and gray shading indicates 100% nucleotide identity between sequences. *bla*_NDM-1_ was embedded in the chromosome of SM_F22R as well as the pSM28_NDM_1 and pSM30_NDM_1, with one, three, and seven tandem copies, respectively. To ensure clear visualization of critical antimicrobial resistance markers (e.g., *bla*_NDM-1_), the IS*CR1* tandem copy structure is represented as n IS*CR1* unit" (where n indicates copy number). The complete plasmid sequences have been deposited in GenBank (pSM28_NDM_1: CP138308.1; pSM30_NDM_1: CP138306.1).

The *bla*_NDM-1_-bearing region in SM_F22R chromosome, SM_F28R plasmid (pSM28_NDM_1, CP138308.1), and SM_B30R plasmid (pSM30_NDM_1, CP138306.1) exhibited high similarity and was demarcated by two IS*26* copies in identical orientation, designated as pTn*7588* (IS*26*-MDR-IS*26*) (Fig. 3). The *bla*_NDM-1_ gene was located downstream of a class 1 integron harboring a cassette array (*dfrA12*) and embedded within the 6,431 bp IS*CR1* unit (IS*CR1-dsbD-trpF-ble-bla*_NDM-1_-IS*Aba125*Δ-IS*1R*-IS*Aba125*Δ) (Fig. 3). Notably, sequence analysis revealed that IS*1R* insertion into a truncated IS*Aba125* element generated a 9 bp TSD (CAACTTGAT), abolishing IS*Aba125* transposition activity.

**Fig 4:**
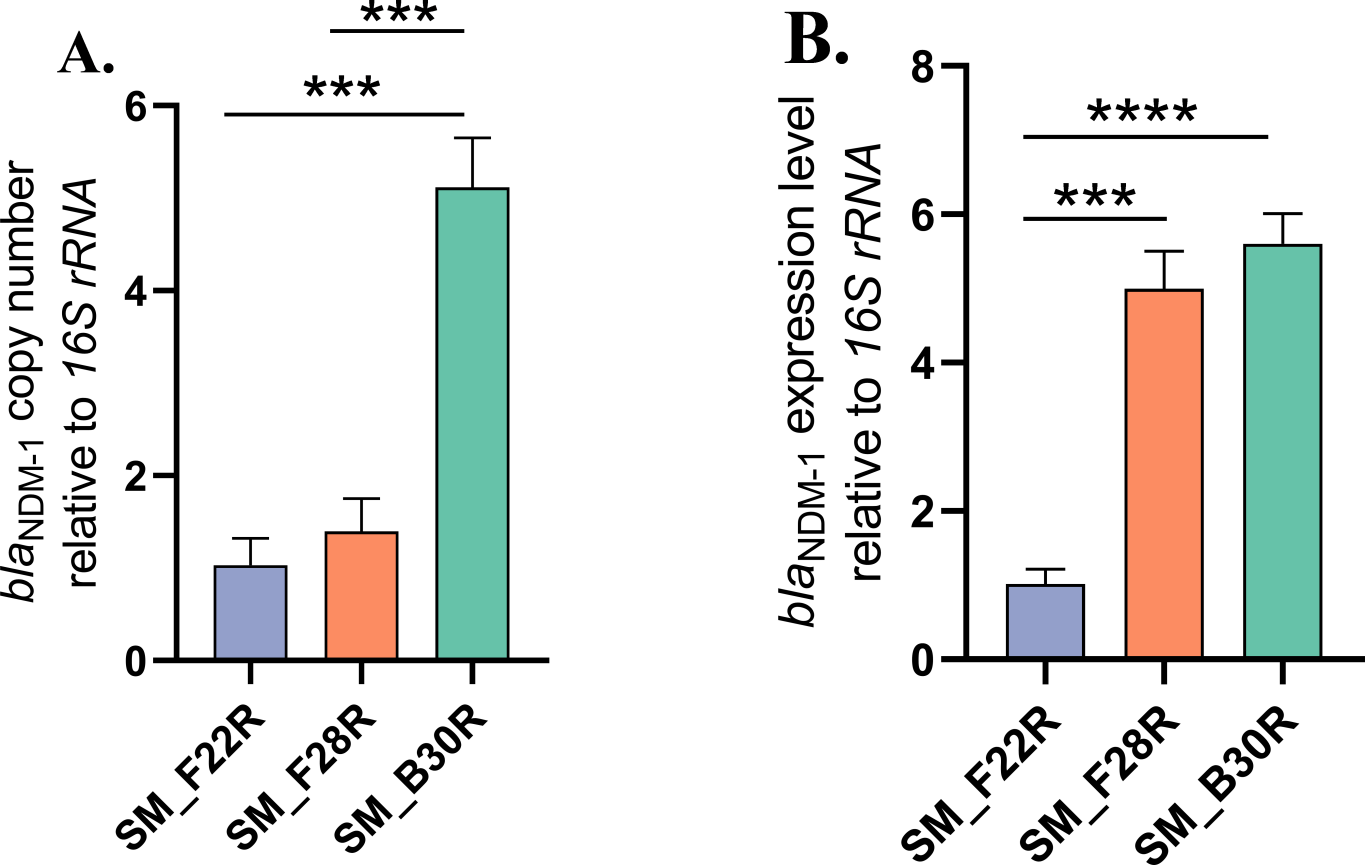
The quantitative real-time PCR results. (**A**) The relative copy number of *bla*_NDM-1_. (**B**) The relative expression level of *bla*_NDM-1_. The *16S rRNA* was used as the reference gene, and SM_F22R as the control group. ***P  <  0.001, ****P  <  0.0001.

pSM_28_NDM_1 and pSM_30_NDM_1 displayed almost identical structures, closely resembling the *Klebsiella pneumoniae* IncC plasmid pKP-14-6-NDM-1 (MN175387) isolated from a 2014 Chinese clinical bloodstream infection. The primary structural variation among these plasmids resided in the differential copy numbers of the IS*CR1* unit, with SM_F22R (chromosomal integration), pSM28_NDM_1, and pSM30_NDM_1 harboring 1, 3, and 7 tandem copies of the IS*CR1* unit, respectively, resulting in different plasmid lengths. Quantitative PCR analysis normalized to *16S rRNA* demonstrated that SM_B30R exhibited significantly elevated *bla*_NDM-1_ copy numbers compared to both SM_F22R and SM_F28R (*P*  <  0.001) (Fig. 4A). Correspondingly, the expression levels of *bla*_NDM-1_ in SM_B30R and SM_F28R were significantly higher than those in SM_F22R (*P*  <  0.001) (Fig. 4B), consistent with the higher MICs of IPM, IMR, MEM, and MEV for SM_B30R and SM_F28R.

### Read depth analysis of *bla*_NDM-1_ and *mph*(A) genes

Given the inherent instability of gene amplification and the technical limitations of sequencing platforms, assembled sequences may not precisely represent true tandem copy numbers. To validate the tandem amplification structures, we performed read mapping analysis by aligning Illumina short reads to the regions carrying *bla*_NDM-1_ and *mph*(A) on pSM_28_NDM_1, pSM_30_NDM_1, and SM_F22R chromosomes. After normalization of sequencing data using *repB* (replication initiator gene of IncC-type plasmid) and *purE* (chromosomal housekeeping gene), we observed that the IS*CR1* unit carrying *bla*_NDM-1_ demonstrated significantly higher coverage of read mapping in SM_B30R compared to SM_F28R (Fig. S4). In both SM_F28R and SM_B30R, the pTn*7682* region containing *mph*(A) exhibited a similar read mapping depth ratio relative to flanking genes. However, SM_F22R exhibited markedly increased read depth ratios across the pTn*7682* region compared to adjacent chromosomal sequences (Fig. S4). Read-depth analysis provided evidence for differential amplification of the IS*CR1* unit and pTn*7682* region among isolates.

## DISCUSSION

*Salmonella* has emerged as a critically important foodborne pathogen with progressively increasing antibiotic resistance profiles, particularly concerning resistance to carbapenems—last-resort antibiotics essential for treating MDR bacterial infections. While previous studies have reported *bla*_NDM-1_-positive *Salmonella* isolates, such as serotypes *Salmonella* London (35), Corvallis (36), and Lomita (37), we represented the first comprehensive characterization of *bla*_NDM-1_-harboring *S*. Mbandaka. Through phenotypic and genomic analysis of five consecutive clinical isolates from a patient progressing from gastrointestinal infection to septicemia, we elucidated the dissemination and amplification mechanisms of *bla*_NDM-1_ mediated by IS*26* and IS*CR1*.

Building upon the previous findings of IS*26*-mediated chromosomal inversions in *Acinetobacter baumannii* through intramolecular replicative transposition (38), we found that the IncC plasmid-bearing isolates (SM_F28R and SM_B30R) evolved from either carbapenem-susceptible precursors (SM_F22S and SM_F28S) or a chromosomally encoded *bla*_NDM-1_ isolate (SM_F22R) via IS*26*-mediated intramolecular replicative transposition. Our findings presented another instance of genomic rearrangements mediated by IS*26*, underscoring its significant role in shaping the genetic landscape of strains.

The amplification of the IS*26-mph*(A)-*mrx*(A)-*mphR*(A)-IS*26* cassette was likely mediated through translocatable unit (TU) excision and reinsertion mechanisms, according to previous reports on the amplification process of other ARGs, such as *bla*_TEM-1B_ (39), *bla*_OXA-1_ (40), *aphA1a* (41), and *bla*_KPC-2_ (42). Notably, our study demonstrated that all five *S*. Mbandaka isolates harboring the *mph*(A) gene (regardless of copy numbers) exhibited azithromycin MICs ≥ 128 mg/L, consistent with the high-level azithromycin resistance phenotype mediated by *mph*(A) (43, 44). From a clinical perspective, extending susceptibility testing beyond 128 mg/L offers limited practical value given that such concentrations are pharmacokinetically unattainable *in vivo*. Furthermore, physicochemical constraints, including antibiotic solubility limitations and the permeability barrier of the Gram-negative bacterial outer membrane, contribute to a resistance plateau phenomenon (45), wherein amplification of ARGs does not consistently correlate with enhanced phenotypic resistance. Based on the above considerations, this study did not contain higher azithromycin concentrations for AST.

The *bla*_NDM_ genes are predominantly located on plasmids, such as IncC, IncX3, IncFII/FI, and IncN2, rather than on the chromosome (46). The *bla*_NDM-1_ gene on the IncC plasmid in our study exhibited a tandem copy structure, with its genetic context differing from previous reports (47, 48). Although in most *bla*_NDM-1_ tandem copy structures, the proximity of IS*CR1* to *bla*_NDM-1_ and its similarity to IS*91* family elements utilizing a rolling-circle mechanism suggest that IS*CR1* may mediate *bla*_NDM-1_ amplification through a similar mechanism, direct experimental evidence for this is still lacking (49). Previous research found that the spontaneous emergence of such tandem amplification occurs at low frequency (~10^5^ CFU) in the absence of antibiotic selection. Namely, it could be difficult to obtain the isolates with the tandem copies without any selection pressures for antibiotics (42). The isolates containing the *mph*(A) and *bla*_NDM-1_ tandem copies in our study originated from a patient with prior exposure to relevant antimicrobial agents (azithromycin and carbapenems), which indicate that such structural variations may be driven by antibiotics. Currently, the understanding of the copy number effect of *bla*_NDM_ remains limited, though transient meropenem resistance enhancement has been reported (50, 51). In our study, SM_F22R, SM_F28R, and SM_B30R demonstrated elevated *bla*_NDM-1_ expression correlating with high levels of carbapenem resistance, but the concurrent variation in both *bla*_NDM-1_ localization (chromosomal vs. plasmid) and copy number precludes definitive assessment of its contribution to the development of resistance.

In our study, carbapenem-resistant and carbapenem-susceptible isolates have been demonstrated to originate from the same clone, but they displayed remarkable differences in their ARGs. The resistant isolates acquired carbapenem resistance through either chromosomal integration of the pTn*7588* carrying *bla*_NDM-1_ or by obtaining an IncC-type plasmid harboring pTn*7588*. In contrast, susceptible isolates lacked both the *bla*_NDM-1_-bearing pTn*7588* and 1,220 kb inversion. The resistant isolates lost the pTn*7588* region on the chromosome or the IncC plasmid in some progeny cells due to unstable replication or asymmetric plasmid distribution during cell division, thereby exhibiting carbapenem susceptibility.

Given the existing chromosomal variants, the integration of pTn*7588* carrying *bla*_NDM-1_ into the chromosome might be explained by the IS*26*-mediated insertion of TU (IS*26*-MDR), which consists of the MDR region carrying *bla*_NDM-1_ and one copy of IS*26*. Although SM_F28R and SM_B30R were isolated later than SM_F22R, we cannot rule out the possibility that plasmid-borne *bla*_NDM-1_ isolates already existed on December 22. Additionally, as the IncC plasmid identified in carbapenem-resistant *S*. Mbandaka isolates is commonly found in public databases, we consider that horizontal transfer of *bla*_NDM-1_ from plasmid to chromosome is the most probable evolutionary route. We propose that the TU could be generated from the IS*26*-flanked pTn*7588* region on the IncC plasmid identified in this study. Once TU (IS*26*-MDR) is inserted into the chromosome, the *bla*_NDM-1_-carrying MDR region flanked by two IS*26* copies becomes pTn*7588*. Previous studies have demonstrated that IS*26* facilitates the transfer of *bla*_NDM-1_ from plasmids to the chromosome via intermolecular replicative transposition (52–54). In our study, the observation of no TSD near pTn*7588* supports that IS*26*-driven *bla*_NDM-1_ transfer may be through a more efficient conservative transposition pathway, which is consistent with its characterization of targeting an existing copy of IS*26* without producing TSD (24, 26). Notably, a study has found that novel transposon Tn*6433* variants facilitate *tet*(E) transfer from chromosome to plasmid in *Aeromonas* under oxytetracycline stress (55). However, the environmental factors driving the chromosomal-plasmid transfer of *bla*_NDM-1_ remain unexplored. Existing research indicates that *bla*_NDM-1_ can be horizontally transferred via outer membrane vesicles (OMVs). Although meropenem exposure increases OMV production, it does not enhance *bla*_NDM-1_ transfer frequency compared to untreated controls (56). Therefore, further research is needed to explore the potential mechanisms that promote *bla*_NDM-1_ mobilization between chromosomes and plasmids.

Our study documented the concurrent presence of *bla*_NDM-1_ in both chromosomal and plasmid locations within *S*. Mbandaka isolates, expanding the host range of *bla*_NDM-1_ and shedding light on its vertical and horizontal transmission mechanisms. The structural variations of regions with ARGs in *S*. Mbandaka provided valuable insights into the dynamic evolutionary processes shaping resistance patterns in this pathogen, highlighting the imperative for adaptive treatment strategies and enhanced infection control protocols to combat resistance dissemination. However, it is important to acknowledge the limitations of our study. While the study proposes a plausible IS*26*-mediated conservative transposition pathway for the transfer of *bla*_NDM-1_ from the plasmid to the chromosome, this pathway could not be experimentally validated, and this study did not discuss the effects of the environmental factors or host antibiotic concentrations on transfer events. Although the single-patient origin of our isolates enabled detailed longitudinal and clonal comparisons, *in vitro* antibiotic induction experiments are still needed in the future, which is essential to validate the generalizability of our findings. Furthermore, the environmental reservoirs of *bla*_NDM-1_-positive *S*. Mbandaka remain undefined, which could inform infection control or surveillance strategies.

### Conclusion

In conclusion, our findings demonstrated that *S*. Mbandaka acquired carbapenem resistance either by chromosomal integration or plasmid acquisition of *bla*_NDM-1_, with *bla*_NDM-1_ amplification contributing to carbapenem resistance enhancement. The identified genetic variations—encompassing IS*26*-mediated chromosomal inversions, *mph*(A) amplification, *bla*_NDM-1_ transfer, and IS*CR1*-driven *bla*_NDM-1_ amplification—collectively demonstrate this pathogen’s remarkable genomic plasticity and adaptive capacity in response to antimicrobial selection pressures and complex host environments.

## Data Availability

All sequencing reads and assemblies were deposited in GenBank under BioProject number PRJNA923735.

## SUPPLEMENTAL MATERIAL

Supplemental Figures spectrum.00967-25-s0001.docx Figures S1 to S4.

ASM does not own the copyrights to Supplemental Material that may be linked to, or accessed through, an article. The authors have granted ASM a non-exclusive, world-wide license to publish the Supplemental Material files. Please contact the corresponding author directly for reuse.
