# Supplementary material for: Emergence of carbapenem-resistant Salmonella Mbandaka through IS26-driven blaNDM-1 mobilization and chromosomal structural variation
Source: Microbiol Spectr. 2025 Aug 15;13(10):e00967-25. doi: 10.1128/spectrum.00967-25 (PMC12502706; doi:10.1128/spectrum.00967-25)
Supplement: Supplemental Figures — Figures S1 to S4. [file spectrum.00967-25-s0001.docx]

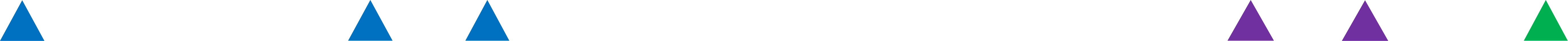


**Supplementary Figure 1.** Clinical progression and antimicrobial susceptibility profiles of *Salmonella* Mbandaka isolates. **A.** Detailed hospital course with treatment timeline. BALF, Bronchoalveolar Lavage Fluid. **B.** The susceptibility test results of isolates to meropenem and imipenem by Kirby-Bauer test. The Kirby-Bauer test results of *Salmonella* Mbandaka isolated on December 22nd and 28th exhibited double inhibition zones when testing carbapenems. Black arrows indicate colonies from the outer zone edge (inhibition zone diameter >23 mm), which were confirmed as carbapenem-susceptible isolates (SM_F22S, SM_F28S). Red arrows indicate colonies from the inner zone edge (inhibition zone diameter <19 mm), which were identified as carbapenem-resistant isolates (SM_F22R, SM_F28R). For the *Salmonella* Mbandaka isolated on December 30th (SM_B30R), only one inhibition zone was observed. The drug on the left side of the Mueller-Hinton agar plates is meropenem, and the drug on the right side is imipenem.

**Supplementary Figure 2.** PFGE and Southern blotting results. **A**. The band profiles of XbaI-PFGE and S1-PFGE. The arrow indicates the difference between SM_F22R and the other four isolates, with an additional band at approximately 210 kb and no band at approximately 33 kb. **B**. The cluster dendrogram of XbaI-PFGE results. **C**. Ladder patterns of Southern blotting using a *bla*_NDM-1_ probe following XbaI-PFGE and S1-PFGE. Lane M, H9812; Lane 1, S1 nuclease-treated SM_F22R; Lane 2, XbaI nuclease-treated SM_F22R; Lane 3, S1 nuclease-treated SM_F28R; Lane 4, S1 nuclease-treated SM_B30R; Arrow, positive signal by Southern blotting hybridization. Lane M, *Salmonella* serotype Braenderup strain H9812 as a molecular marker.

**Supplementary Figure 3.** Schematic representation of genetic variations of the chromosome. The genetic variations in the chromosome of five *Salmonella* Mbandaka isolates include an inversion of a ~1,220 kb region and an insertion of a ~26 kb IS*26* region (carrying *bla*_NDM-1_). In addition, this figure shows the XbaI recognition sites on chromosomes and the size of DNA fragments generated by simulating XbaI digestion of the genome. The diagram is not to scale. The gray shading between chromosome sequences indicates a 100% nucleotide identity. The blue shading between chromosome sequences indicates a 1,220 kb reversion region. The genes are represented by corresponding colored arrows, and the gene names are displayed on the graph. The yellow flag facing right represents the 8 bp sequence 5'-ATTTTGCG-3', while the yellow flag facing left represents the 8 bp sequence 5'-CGCAAAAT-3'.


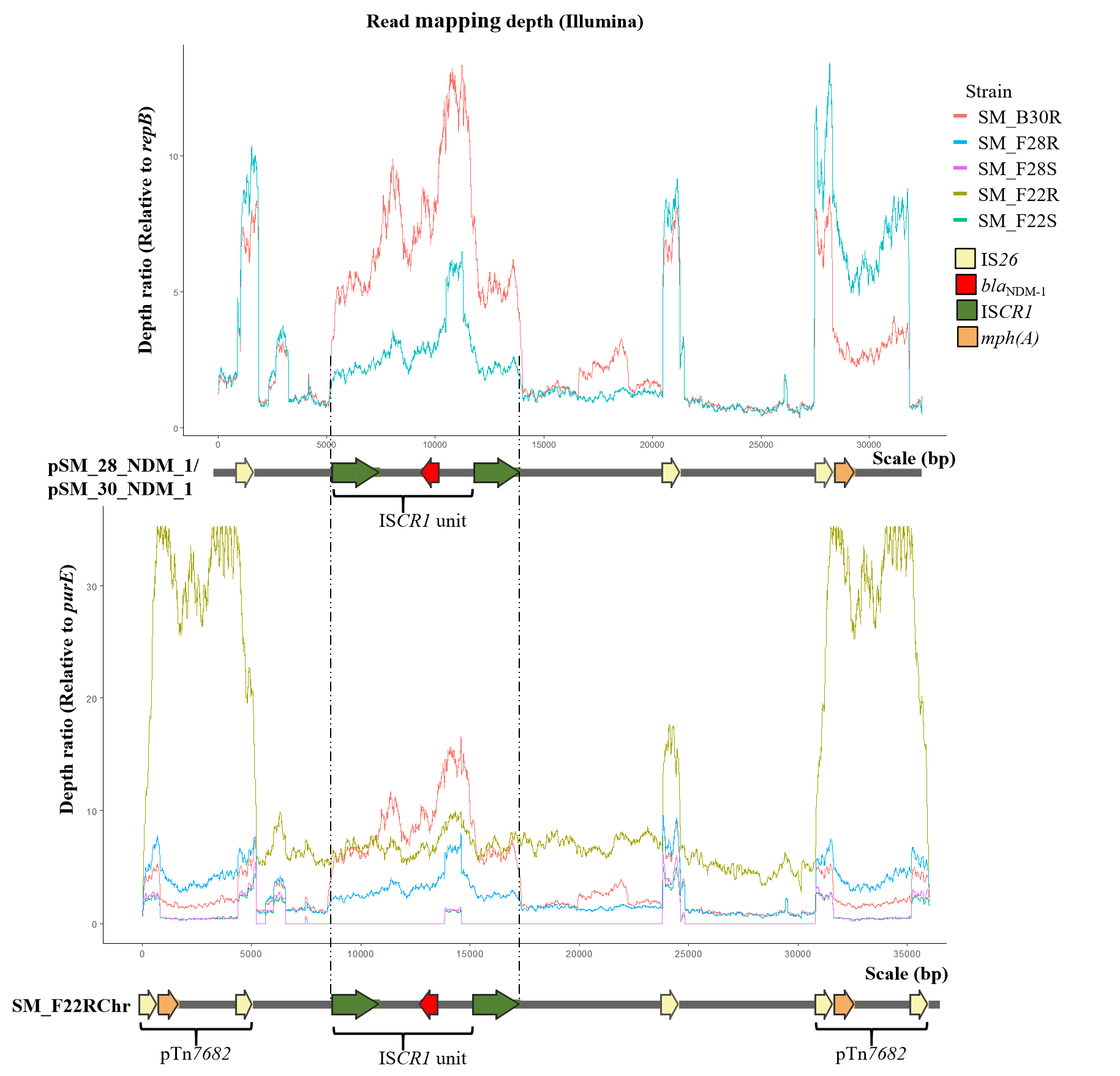


**Supplementary Figure 4.** Read mapping depth of Illumina sequencing short reads to the regions carrying *bla*_NDM-1_ and *mph*(A) on pSM_28_NDM_1, pSM_30_NDM_1, and SM_F22R chromosome. Read mapping depths were normalized to the IncC-type plasmid replication initiator gene *repB* and the chromosome housekeeping gene *purE*, respectively.
